# Dynamics and prognostic role of galectin-3 in patients with advanced heart failure, during left ventricular assist device support and following heart transplantation

**DOI:** 10.1186/s12872-016-0298-z

**Published:** 2016-06-14

**Authors:** Ellie Coromilas, Em-Claire Que-Xu, D’Vesharronne Moore, Tomoko S. Kato, Christina Wu, Ruiping Ji, Raymond Givens, Ulrich P. Jorde, Hiroo Takayama, Yoshifumi Naka, Isaac George, Donna Mancini, P. Christian Schulze

**Affiliations:** Department of Medicine, Division of Cardiology, Columbia University Medical Center, 166 Fort Washington Avenue, New York, NY 10032 USA; Division of Cardiothoracic Surgery, Department of Surgery, Columbia University Medical Center, 166 Fort Washington Avenue, New York, NY 10032 USA; Department of Medicine, Division of Cardiology, Angiology, Pneumology and Intensive Medical Care, Friedrich-Schiller-University Jena, Erlanger Allee 101, 07743 Jena, Germany

**Keywords:** Heart Failure, Galectin-3, LVAD, Heart Transplantation, Coronary Allograft Vasculopathy

## Abstract

**Background:**

Galectin-3 is a marker of myocardial inflammation and fibrosis shown to correlate with morbidity and mortality in heart failure (HF). We examined the utility of galectin-3 as a marker of the severity of HF, the response of galectin-3 levels to ventricular assist device (LVAD) implantation or heart transplantation (HTx), and its use as a prognostic indicator.

**Methods:**

Plasma galectin-3 was measured using a commercially available ELISA assay in patients with stable HF (*n* = 55), severe HF (*n* = 63), at 3 (*n* = 17) and 6 (*n* = 14) months post-LVAD and at LVAD explantation (*n* = 23), patients following HTx (*n* = 85) and healthy controls (*n* = 30).

**Results:**

Galectin-3 levels increase with the severity of HF (severe HF: 28.2 ± 14, stable HF: 19.7 ± 13, *p* = 0.001; controls: 13.2 ± 9 ng/ml, *p* = 0.02 versus stable HF). Following LVAD implantation, galectin-3 levels are initially lower (3 months: 23.7 ± 9, 6 months: 21.7 ± 9 versus 29.2 ± 14 ng/ml implantation; *p* = NS) but are higher at explantation (40.4 ± 19 ng/ml; *p* = 0.005 versus pre-LVAD). Galectin-3 levels >30 ng/ml are associated with lower survival post-LVAD placement (76.5 % versus 95.0 % at 2 years, *p* = 0.009). After HTx, galectin-3 levels are lower (17.8 ± 7.1 ng/ml post-HTx versus 28.2 ± 14 pre-HTx; *p* < 0.0001). Patients with coronary allograft vasculopathy (CAV) post-HTx showed higher galectin-3 levels (20.5 ± 8.8 ng/ml versus 16.8 ± 6.3, *p* = 0.1) and the degree of CAV correlated with levels of galectin-3 (*r*^2^ = 0.17, *p* < 0.0001).

**Conclusions:**

Galectin-3 is associated with the severity of HF, exhibits dynamic changes during mechanical unloading and predicts survival post-LVAD. Further, galectin-3 is associated with the development on CAV post-HTx. Galectin-3 might serve as a novel biomarker in patients with HF, during LVAD support and following HTx.

## Background

The syndrome of chronic heart failure (HF) is associated with increasing morbidity and mortality throughout the world. As the failing heart deteriorates in function, ventricular dilatation and hypertrophy compensate for increased wall stress associated with myocardial inflammation and cardiac fibrosis. Proliferating myofibroblasts deposit pro-collagen I into the myocardial matrix which is cross-linked to form collagen I [1–3]. Fibrotic remodeling and associated collagen deposition results in myocardial tissue heterogeneity and increased stiffness contributing to a vicious cycle of progressive cardiac dysfunction [2, 3].

Galectin-3, a paracrine factor secreted by macrophages, has been identified as a critical participant in the pathogenesis and progression of cardiac fibrosis and inflammation [2, 3]. Galectin-3 is secreted in response to mechanical stress and neurohormonal stimuli and potentiates TGF-β signaling, a critical regulator of cardiac fibrosis [2]. Therefore, galectin-3 is a particularly intriguing biomarker. Unlike current indicators of HF severity, it is directly implicated in the pathogenesis of cardiac fibrosis. Recent studies have shown that galectin-3 correlates with HF severity and may be predictive of clinical outcomes in HF patients [4–7].

Cardiac fibrosis directly correlates with the degree of ventricular dysfunction and dilatation as well as with myocardial wall stress. The impact of mechanical unloading through left ventricular assist device (LVAD) implantation on myocardial fibrosis is controversial and might be influenced by the type of LVAD implanted, underlying cardiomyopathy, and HF duration [8]. Nevertheless, LVAD implantation has evolved into a standard therapy for patients with advanced HF, serving either as destination therapy or as a “bridge-to-transplantation” [9]. Notably, restoration of cardiac output with LVADs has been shown to reverse cardiomyocyte hypertrophy and decrease ventricular end-diastolic dimensions [8, 10].

Interstitial myocardial fibrosis has also been linked to cardiac remodeling following heart transplantation (HTx) and in particular to the development of cardiac allograft vasculopathy (CAV). CAV is a largely immune-mediated process characterized by diffuse neo-intimal proliferation leading to coronary artery stenosis, which is a major cause of morbidity and mortality in patients following HTx with limited treatment options. Ten percent of HTx recipients are diagnosed with CAV 1 year post-HTx and more than 50 % have CAV by 10 years post-HTx [11].

We hypothesized that galectin-3 levels correlate with severity of HF and respond to mechanical unloading through LVAD placement. Therefore, we analyzed galectin-3 levels in patients with various degrees of HF, before and after mechanical unloading through LVAD implantation, and following HTx.

## Methods

### Study design and patient population

We included 118 patients with HF in this study. Fifty-five patients had stable HF defined as NYHA Class of I-IIIA. Sixty-three patients had severe HF (NYHA Class IIIB-IV), and 57 of these patients received LVADs either as a bridge-to-transplantation or destination therapy. Of the patients who received LVADs, 52 received a Heartmate II axial flow LVAD (Thoratec, Pleasanton, CA) and 5 received a Heartware centrifugal flow LVAD (Heartware, Framingham, MA). Follow-up samples were collected after device implantation (17 patients at 3 months post-LVAD and 14 patients at 6 months). Of the 57 patients who received LVADs, 14 had their LVADs explanted during HTx. Nine additional patients were recruited at the time of LVAD explantation, creating a LVAD-explantation cohort of 23 patients. In addition, 85 patients following HTx (1 month to 20 years post-HTx) were analyzed. All patients were evaluated for the presence of CAV by invasive coronary angiogram. CAV was defined according to the ISHLT CAV grading system. Twenty-three patients were diagnosed with CAV and 62 patients showed no sign of CAV. There were 30 healthy subjects who served as controls and were selected to match the age and gender of patients in the other cohorts. Patients with significant medical history, including but not limited to heart disease, lung disease, kidney disease, liver disease, cancer, stroke, diabetes, and thyroid disorders were excluded.

This is a retrospective study utilizing samples obtained from HF patients at Columbia University, some of whom underwent LVAD implantation or heart transplantation. All blood samples were immediately processed by centrifugation for collection of plasma and serum. Plasma was transferred to 1 mL cryotubes and stored at −80 °C until analysis. Clinical, laboratory, and outcome information was collected from patient medical records. All patients were recruited from the clinics or inpatient services at Columbia University Medical Center. The study protocol was approved by the Institutional Review Board at Columbia University Medical Center and written informed consent was obtained from all individuals prior to inclusion into the study.

### Galectin-3 measurements

Plasma galectin-3 was measured using a commercially available ELISA assay (Galectin-3 Assay, BG Medicine, Waltham, MA), which has a measuring range of 1.4 to 94.8 ng/mL and coefficient of variation of 10.4 %, with no significant cross reactivity. Samples were run on a SpectraMax 250 microplate reader (Molecular Devices, Sunnyvale, CA). Calibration of the assay was performed according to the manufacturer’s recommendations and values were normalized to a standard curve.

### Statistical analyses

Data were presented as mean ± standard deviation. Comparisons among all groups were performed using ANOVA or Students’s *t*-test as appropriate, as well as correlational analysis using Spearman’s correlation. ROC analysis was used to identify galectin-3 levels predictive of poor outcome and event rates were calculated using the Kaplan-Meier method. Probability values of *p* < 0.05 were considered to be statistically significant.

## Results

### Galectin-3 levels in patients with different degrees of heart Failure

Galectin-3 levels were measured in 55 patients with stable HF (defined as NYHA Class I-IIIA), 63 patients with severe HF (NYHA Class IIIB-IV) and 30 healthy controls. Baseline demographics are listed in Table 1. Patients with severe HF had lower LVEF and higher BNP levels compared to those with stable HF. Patients with severe HF were also less likely to be hypertensive, and more likely to have concurrent kidney disease.

**Table 1:** Demographic data of patients with HF

	Controls (*n* = 30)	Stable HF (*n* = 55)	*p*-value*	Severe HF (*n* = 63)	*p*-value†
Galectin-3 (ng/ml)	13.2 ± 9	19.7 ± 13	0.02	28.2 ± 14	0.001
Age (yrs)	52 ± 8	59 ± 10	0.001	62 ± 11	NS
Gender (% male)	73 %	76 %	NS	92 %	0.01
BMI (kg/m^2^)	27 ± 5	28 ± 4	NS	28 ± 5	NS
Etiology (%)					
Ischemic	-	45 %	-	56 %	NS
Dilated	-	42 %	-	40 %	
Hypertrophy	-	2 %	-	1 %	
Unspecified	-	11 %	-	3 %	
NYHA Class	-	2.3 ± 0.8	-	3.7 ± 0.4	<0.0001
LVEF (%)	-	24 ± 9	-	18 ± 5	<0.0001
BNP (ng/mol)	-	707 ± 762	-	1384 ± 1097	0.001
Comorbidities					
Hypertension	13.3 %	61.8 %	<0.0001	49.2 %	0.007
Previous MI^a^	0 %	41.8 %	<0.0001	38.1 %	NS
Hyperlipidemia	23.3 %	50.9 %	0.0005	41.2 %	NS
Diabetes	0 %	30.9 %	0.0001	39.7 %	NS
Kidney Disease	0 %	12.7 %	0.02	38.1 %	0.007
COPD^b^	0 %	9.1 %	0.04	14.3 %	NS
Smoker	6.7 %	5.5 %	NS	1.6 %	NS
Lab Values					
WBC^c^ (x10^3^/μL)	-	7.1 ± 2	-	8.8 ± 3	0.001
Hct^d^ (g/dL)	-	39 ± 4	-	32 ± 10	<0.0001
Na^e^ (mEq/L)	145 ± 10	137 ± 3	<0.0001	135 ± 3	0.0008
Creat^f^ (mg/dL)	1.0 ± 0.3	1.3 ± 0.9	NS	1.5 ± 0.5	NS
T-Bili^g^ (mg/dL)	0.5 ± 0.2	0.7 ± 0.4	0.007	1.6 ± 1.4	0.0004
Albumin (g/dL)	4.9 ± 0.4	4.3 ± 0.4	<0.0001	3.6 ± 0.5	<0.0001

*p value versus Controls †*p* value versus Stable HF

^a^ *MI* myocardial infarction, ^b^ *COPD* chronic obstructive pulmonary disease, ^c^ *WBC* white blood count, ^d^ *Hct* hematocrit, ^e^ *Na* sodium, ^f^ *Crea* creatinine, ^g^ *T*-*Bili* total bilirubin

**Fig. 1:**
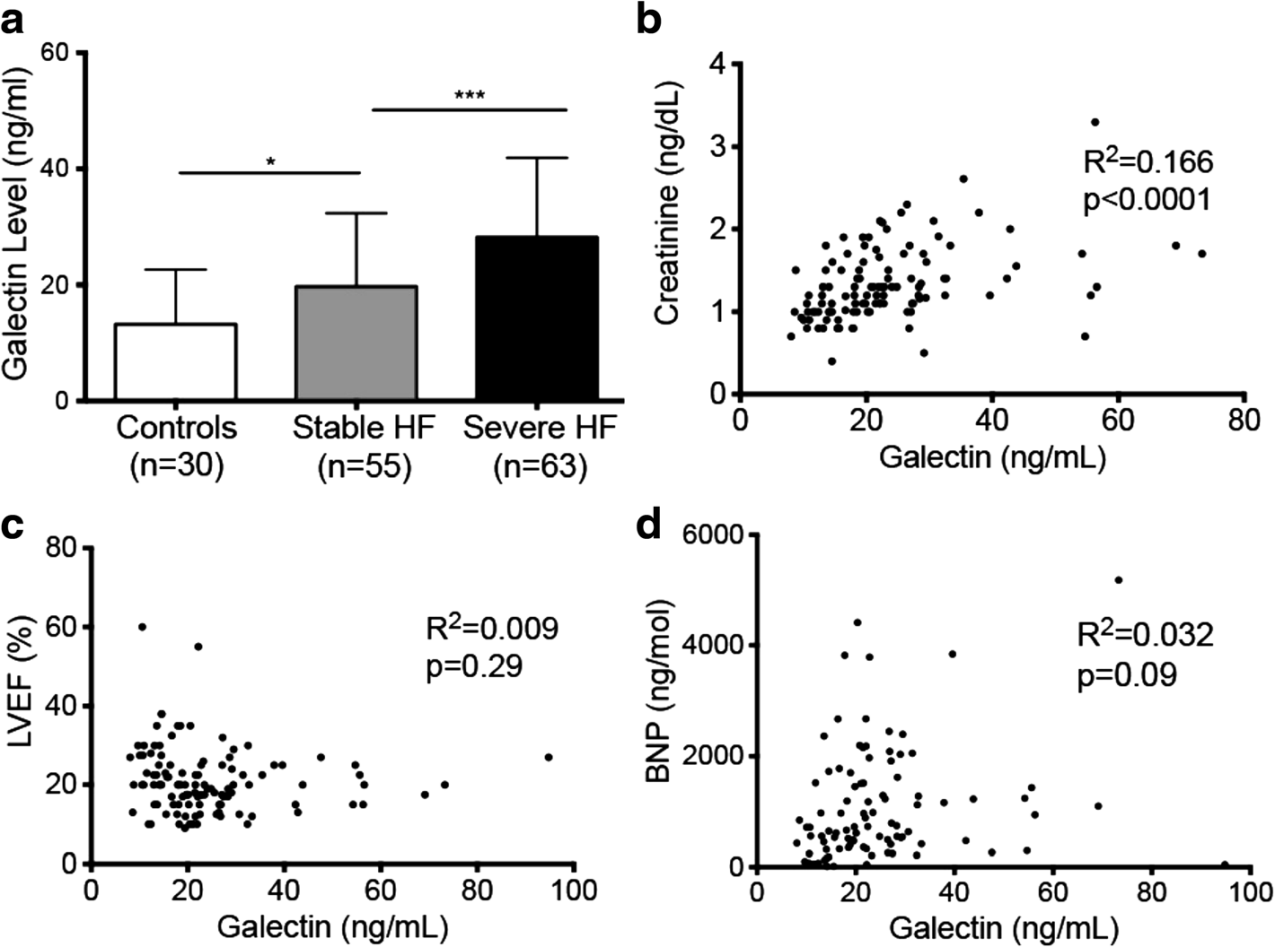
Galectin-3 levels correlate with severity of HF. **a** Stepwise increase in mean galectin-3 levels in patients with increasing severity of HF (*p < 0.05; ***p < 0.001). **b** Galectin-3 levels correlate with creatinine levels in patients with HF and controls. **c** Lack of correlation of galectin-3 with mean LVEF. **d** Lack of correlation of galectin-3 with BNP

Patients with stable HF had higher galectin-3 levels compared to healthy controls (19.7 ± 13 versus 13.2 ± 9 ng/ml; *p* = 0.02). Galectin-3 levels were higher in patients with severe HF compared to patients with stable HF (28.2 ± 14 ng/ml in severe HF; *p* = 0.001) (Fig. 1a).

Galectin-3 levels correlated with severity of HF as defined by NYHA class (*p* = 0.001) and creatinine (*R*^2^ = 0.166; *p* < 0.0001) (Fig. 1b). There was no correlation between galectin-3 levels and BNP (*R*^2^ = 0.032; *p* = 0.09) or LVEF (*R*^2^ = 0.0095; *p* = 0.30) (Fig. 1c-d)

### Dynamics of galectin-3 levels following LVAD implantation

Of the 57 patients who received LVADs, 17 patients had follow-up levels measured at 3-months, 14 patients had follow-up levels measured at 6-months and 14 patients had levels measured at the time of LVAD explantation. An additional 9 patients were analyzed at the time of explantation. On average, patients who underwent LVAD explantation had been on LVAD support for 277 ± 223 days. Demographic information of this cohort at baseline and during follow-up is listed in Table 2.

**Table 2:** Demographic data of patients undergoing LVAD placement

	LVAD Implant (*n* = 57)	3 Months Post-LVAD (*n* = 17)	*p*-value*	6 Months Post-LVAD (*n* = 14)	*p*-value*	LVAD Explant (*n* = 23)	*p*-value*
Galectin-3 (ng/ml)	29.2 ± 14	23.7 ± 8	NS	21.7 ± 9	NS	40.4 ± 19	0.005
Age (yrs)	63 ± 10	65 ± 9	NS	60 ± 12	NS	63 ± 8	NS
Gender (% male)	91 %	100 %	NS	93 %	NS	87 %	NS
BMI (kg/m^2^)	28 ± 5	28 ± 4	NS	28 ± 5	NS	26 ± 4	NS
Etiology (%)							
Ischemic	56 %	65 %	NS	64 %	NS	65 %	NS
Dilated	39 %	35 %		36 %		35 %	
Hypertrophy	2 %	0 %		0 %		0 %	
Unspecified	3 %	0 %		0 %		0 %	
BNP (ng/mol)	1378 ± 1084	-	-	-	-	737 ± 470	0.01
LVAD Type (%)							
Continuous	91 %	100 %	NS	82 %	NS	100 %	NS
Pulsatile	9 %	0 %		18 %		0 %	
Comorbidities							
Hypertension	54.4 %	64.7 %	NS	42.9 %	NS	47.8 %	NS
Previous MI^a^	42.1 %	47.1 %	NS	35.7 %	NS	52.2 %	NS
Hyperlipidemia	45.6 %	52.9 %	NS	42.9 %	NS	47.8 %	NS
Diabetes	39.7 %	52.9 %	NS	21.4 %	NS	30.4 %	NS
Kidney Disease	38.1 %	64.7 %	NS	35.7 %	NS	43.5 %	NS
COPD^b^	15.8 %	29.4 %	NS	7.1 %	NS	17.4 %	NS
Smoker	1.8 %	0 %	NS	0 %	NS	4.4 %	NS
Lab Values							
WBC^c^ (x10^3^/μL)	9.0 ± 3	9.4 ± 4	NS	8.9 ± 4	NS	9.3 ± 4	NS
Hct^d^ (g/dL)	31 ± 10	32 ± 9	NS	35 ± 7	NS	30 ± 4	NS
Na^e^ (mEq/L)	136 ± 4	136 ± 4	NS	135 ± 4	NS	137 ± 4	NS
Crea^f^ (mg/dL)	1.5 ± 0.5	1.6 ± 0.4	NS	1.4 ± 0.5	NS	1.3 ± 0.6	NS
T-Bili^g^ (mg/dL)	1.6 ± 1.4	1.3 ± 0.4	NS	1.5 ± 1.7	NS	1.2 ± 0.9	NS
Albumin (g/dL)	3.6 ± 0.5	3.5 ± 0.5	NS	3.8 ± 0.4	NS	3.3 ± 0.5	0.02

*p value versus LVAD Implant

^a^ *MI* myocardial infarction, ^b^ *COPD* chronic obstructive pulmonary disease, ^c^ *WBC* white blood count, ^d^ *Hct* hematocrit, ^e^ *Na* sodium, ^f^ *Crea* creatinine, ^g^ *T*-*Bili* total bilirubin

Hemodynamic unloading was associated with lower BNP levels following LVAD treatment (1378 ± 1084 versus 737 ± 470 pmol/L at explant, *p* = 0.01). Further, LVAD support was associated with improvement in echocardiographic parameters with a smaller left ventricular end-diastolic diameter (LVEDD) and left atrial (LA) diameter (LVEDD: 67.2 ± 10 versus 58.4 ± 10 mm at explant, *p* = 0.012; LA: 50.1 ± 60 versus 44.3 ± 70 mm at explant, *p* = 0.004). There was also a trend towards smaller left ventricular end systolic diameter (LVESD: 60.8 ± 11 versus 52.8 ± 14 mm, *p* = 0.1). There was no significant change in aortic root diameter (3.4 ± 0.4 vs. 3.5 ± 0.3 cm; *p* = 0.39).

**Fig. 2:**
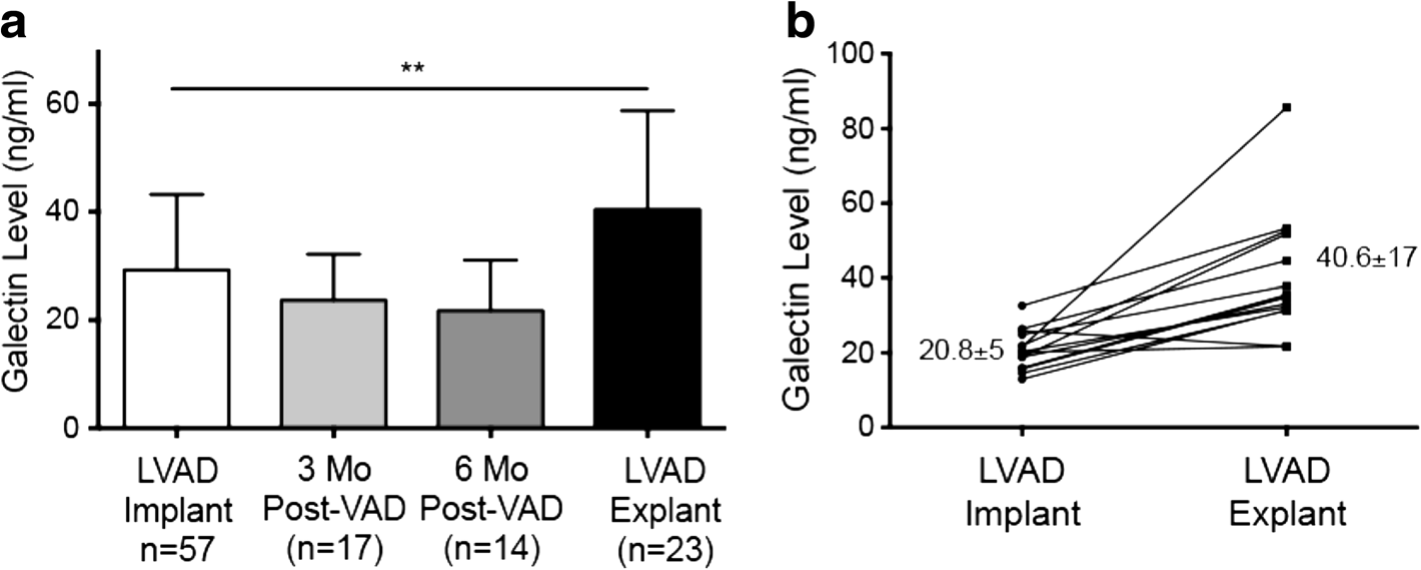
Changes in galectin-3 levels following LVAD implantation. **a** Galectin-3 levels show a trend towards lower levels early after LVAD placement at 3 and 6 months but were higher in patients at the time of LVAD explanation (**p < 0.01). **b** Dynamics of 14 paired patient samples time of implantation to time of explantation

Following LVAD implantation, a downward trend in galectin-3 levels was noted, although it did not reach significance (29.2 ± 14 ng/ml at implantation versus 23.7 ± 8 ng/ml 3 months post-implantation; *p* = 0.13) (Fig. 2a). Six months post-implantation, the average galectin-3 level was 21.7 ± 9 ng/ml (versus 29.2 ± 14 ng/ml at implantation, *p* = 0.06). Of the 17 patients who were evaluated 3 months following implantation, 64.7 % had lower galectin-3 levels compared to implantation. For these 17 patients, the mean galectin-3 level was lower at 3 months, though this difference was not significant (23.7 ± 8 at 3 months versus 29.0 ± 14.5 ng/ml at implantation, *p* = 0.2). Of the 14 patients who were evaluated at 6 months, 57.1 % had lower galectin-3 levels compared implantation. Again, the mean was lower at 6 months for these patients than at the time of implantation (21.7 ± 9 ng/ml at 6 months versus 28.1 ± 15 ng/ml at implantation, *p* = 0.18).

Surprisingly, galectin-3 levels were significantly higher at the time of LVAD explantation (40.4 ± 19 at explant versus 29.2 ± 14 ng/ml at the time of implantation, *p* = 0.005). Of the 23 explanted patients, 14 patients were evaluated both at implantation and explantation, allowing for direct comparison of values. Of these 14 patients, 13 (92.9 %) had elevated galectin-3 levels at the time of explantation as compared to time of implantation (Fig. 2b). Galectin-3 was significantly higher at the time of explantation compared to implantation in these 14 patients. (40.6 ± 17 versus 20.8 ± 5 ng/ml, *p* = 0.0002).

### Prognostic impact of galectin-3 levels on clinical outcomes

**Fig. 3:**
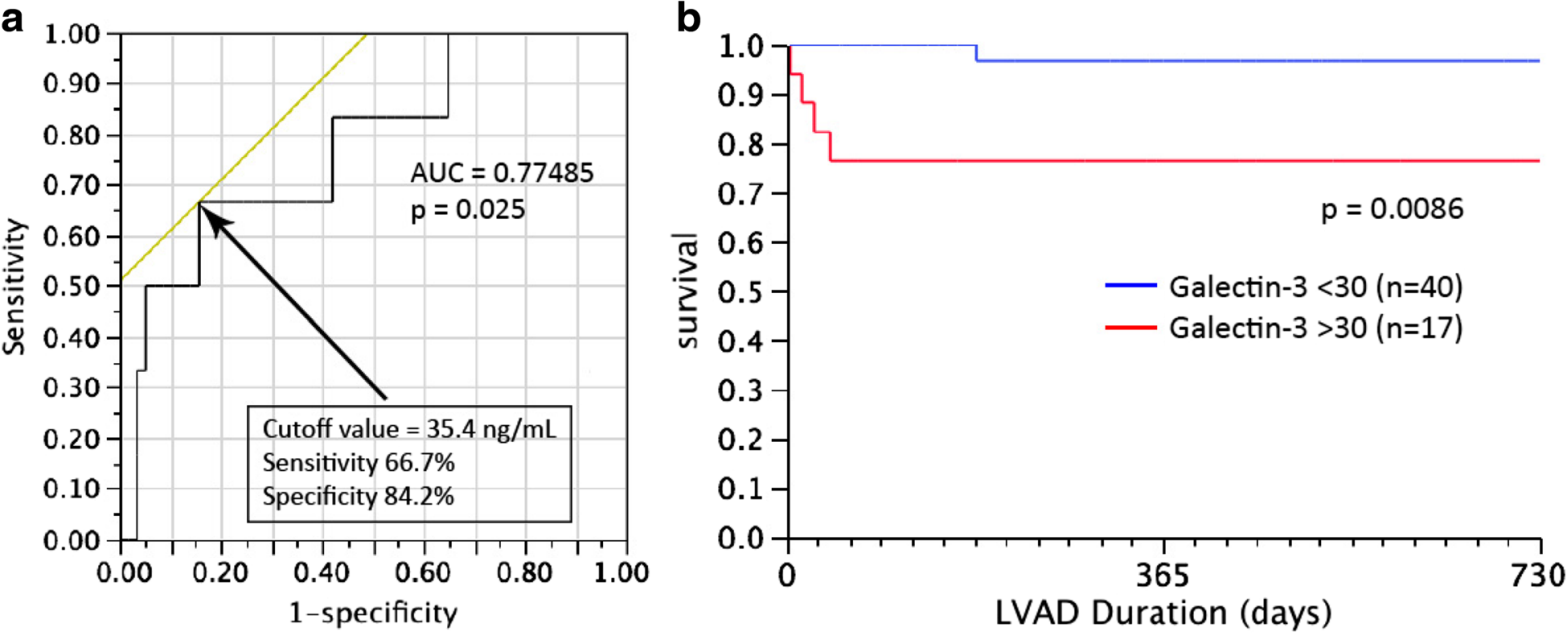
Pre-implant galectin-3 levels predict postoperative mortality following LVAD placement. **a** ROC analysis of patients with severe HF revealed a cut-off value of 35.4 ng/ml as predictive of poor outcome (sensitivity of 66.7 % and specificity of 84.2 %, *p* = 0.025). **b** Patients with galectin-3 level >30 ng/ml at the time of LVAD implantation showed higher mortality during the first 2 years post-LVAD implantation (log rank *p* = 0.0086)

Patients with severe HF who died during the study had significantly higher galectin-3 levels than those who were still alive (48.6 ± 16 versus 28.2 ± 14 ng/ml, *p* = 0.02). To determine if galectin-3 could be used as a prognostic indicator in this cohort, we performed an ROC curve analysis of patients with severe HF and determined that a pre-operative galectin-3 cutoff value of 35.4 ng/ml predicted higher mortality following LVAD placement (66.7 % specificity, 84.2 % sensitivity, *p* = 0.025) (Fig. 3a). Given this, we chose a clinically useful cutoff of 30 ng/ml and dichotomized patients based on this level. Galectin-3 levels greater than 30 ng/ml at the time of LVAD implantation are associated with poor survival at 2 years post implantation (*p* = 0.009) (Fig. 3b). 2 years following implantation, 95.0 % of patients with galectin-3 levels <30 ng/ml at the time of LVAD implantation were still alive, compared to 76.5 % of patients with galectin-3 levels >30 ng/ml at implantation.

### Galectin-3 levels decrease following heart transplantation

**Fig. 4:**
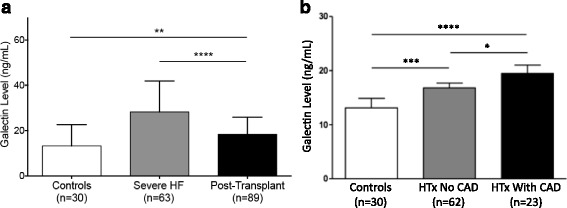
Changes in galectin-3 levels following HTx. **a** Levels of galectin-3 are higher in patients with HF and near normal levels in patients following HTx. **b** Higher circulating levels of galectin-3 following HTx and in the presence of CAV (*p < 0.05; **p < 0.001; ****p < 0.0001)

We analyzed galectin-3 levels in 85 patients following HTx (1 month-20 years post-HTx, Table 3). On average, galectin-3 levels were measured 5.4 ± 0.64 years post-HTx. Galectin-3 levels were lower following HTx compared to in patients with severe HF (17.8 ± 7.3 post-HTx versus 28.2 ± 14 ng/ml in severe HF, *p* < 0.0001) (Fig. 4a). However, galectin-3 levels post-HTx were found to be higher compared to healthy controls (17.8 ± 7.3 versus 13.2 ± 9.4 ng/ml in controls, *p* = 0.002) and comparable to levels in patients with stable HF (19.7 ± 13.0 ng/ml in stable HF, *p* = 0.44). No correlation was found between galectin levels and time since HTx.

**Table 3:** Demographic data of patients following HTx

	Control (*n* = 30)	Post-HTx (*n* = 85)	*p*-value*	No CAV (*n* = 62)	CAV (*n* = 23)	*p*-value†
Galectin-3 (ng/ml)	13.2 ± 9	17.8 ± 7	0.002	16.8 ± 6	20.5 ± 9	0.03
Age (yrs)	52 ± 8	55 ± 14	NS	54 ± 14	58 ± 13	NS
Gender (% male)	73 %	71 %	NS	80 %	81 %	0.02
BMI (kg/m^2^)	27 ± 5	29 ± 6	NS	29 ± 6	28 ± 5	NS
Etiology (%)						
Ischemic	-	32 %	-	54 %	45 %	NS
Dilated	-	43 %		31 %	45 %	
Hypertrophy	-	2 %		0 %	3 %	
Unspecified	-	13 %		15 %	7 %	
LVEF (%)	-	59 ± 8		60 ± 8	55 ± 8	<0.0001
Years post-HTx	-	5.4 ± 0.6	-	5.2 ± 1.2	5.5 ± 1.0	NS
Comorbidities						
Hypertension	13.3 %	61.7 %	<0.0001	55.6 %	67.7 %	NS
Prior MI^a^	0 %	32.6 %	0.0003	25.9 %	45.2 %	NS
Hyperlipidemia	23.3 %	48.3 %	0.02	50.0 %	45.2 %	NS
Diabetes	0 %	49.4 %	<0.0001	50.0 %	41.9 %	NS
Kidney Disease	0 %	38.2 %	<0.0001	31.5 %	48.4 %	NS
COPD^b^	0 %	6.7 %	NS	7.4 %	3.2 %	NS
Smoker	6.7 %	5.6 %	NS	7.4 %	3.2 %	NS
Lab Values						
WBC^c^ (x10^3^/μL)	7.6 ± 5	6.8 ± 2.3	NS	6.9 ± 2.2	6.6 ± 2.6	NS
Na^d^ (mEq/L)	145 ± 10	139 ± 2	<0.0001	139 ± 2	140 ± 2	NS
Crea^e^ (mg/dL)	1.0 ± 0.3	1.5 ± 0.6	<0.0001	1.5 ± 0.5	1.7 ± 0.6	NS
T-Bili^g^ (mg/dL)	0.5 ± 0.2	0.7 ± 0.4	0.01	0.7 ± 0.3	0.8 ± 0.4	NS
Albumin (g/dL)	4.9 ± 0.4	4.3 ± 0.4	<0.0001	4.3 ± 0.4	4.2 ± 0.5	0.05

*p value versus controls; †*p* value versus no CAV

^a^ *MI* myocardial infarction, ^b^ *COPD* chronic obstructive pulmonary disease, ^c^ *WBC* white blood count, ^e^ *Na* sodium, ^f^ *Crea* creatinine; ^g^ *T*-*Bili* total bilirubi006E

Patients following HTx were sub-classified with regard to the occurrence of CAV according to the ISHLT CAV grading system (CAV Grade 0: *n* = 62, Grade 1–3: *n* = 23). Galectin-3 levels were higher in patients with evidence of CAV compared to those without CAV (20.5 ± 8.9 versus 16.8 ± 6.3 ng/mL without CAV, *p* = 0.03) (Fig. 4b). Spearman correlational analysis revealed a correlation between galectin-3 levels and the occurrence of CAV (*r*^2^ = 0.17, *p* < 0.0001).

## Discussion

In this study, we measured galectin-3 levels in patients with various stages of HF, following LVAD support, and after HTx. Galectin-3 was higher in advanced stages of HF compared to controls, was lower post-LVAD, but increased at the time of LVAD explantation. Baseline galectin-3 greater than 30 ng/mL predicted poor outcome following LVAD placement. Patients following HTx on average have lower galectin-3 levels, but levels are higher in the presence of CAV.

Interest in galectin-3 as a potential marker of the severity and outcome of HF has increased with evidence that it is involved in the pathogenesis of cardiac fibrosis and HF progression. By stimulating TGF-β, galectin-3 potentiates collagen deposition and extracellular matrix turnover, leading to an increased proportion of stiffer collagen I in the failing myocardium [12–14]. Prior studies have identified galectin-3 as the most robustly up-regulated protein in models of cardiac hypertrophy and left ventricular dysfunction, and mice with a galectin-3 gene deletion develop myocardial hypertrophy without cardiac dysfunction and fibrosis [5, 15]. Consistent with these animal models, galectin-3 levels have consistently been shown to correlate with disease severity and NYHA class in HF [3, 6, 7].

Our results confirmed that galectin-3 levels correlated with the severity of HF, and levels in healthy subjects were consistent with prior reports defining the normal range as less than 18 ng/ml [7]. Although BNP and LVEF are often used to measure HF severity, we found that galectin-3 levels were not directly associated with these parameters, though some prior studies have shown correlation between galectin-3 and these measures. [16]. Because the majority of HF patients in our study had ischemic HF, our results should only be applied to this population, although others have shown comparable levels of galectin-3 in patients with ischemic and non-ischemic HF [5].

### Galectin-3 levels with LVAD support

That galectin-3 levels may initially decrease after LVAD implantation but were significantly increased at explantation offers interesting insight into the pathologic changes that occur with mechanical support. Evidence regarding changes in fibrosis following LVAD implantation have been conflicting [17]. Our group previously demonstrated decreased ECM turnover with reduced levels of circulating and myocardial metalloproteinases following LVAD placement [18]. Similarly, others have shown decreased myocardial collagen content in LVAD-supported patients [19, 20]. However, others have shown an increase in collagen deposition and an associated increase in myocardial stiffness [10, 21, 22].

Only one recent study analyzed changes in galectin-3 levels in patients undergoing LVAD implantation. Milting et al. found that 30 days post-implantation, ECM turnover defined by changes in levels of matrix metalloproteinases and their tissue inhibitors increased, but galectin-3 levels remained the same [23]. Our average length of LVAD treatment of 277 days is longer than in the study by Milting et al., which may explain why we were able to detect an increase in galectin-3 levels.

The dynamics of galectin-3 levels following LVAD implantation may be indicative of the complicated changes in myocardial structure and fibrosis, and further studies are necessary to more clearly elucidate these fluctuations in galectin-3 levels. Because galectin-3 is secreted in response to mechanical stress, decreased expression shortly after device implantation may be explained by ventricular unloading. However, elevated levels at explantation may indicate that prolonged mechanical support leads to an increase in inflammation and cardiac fibrosis. This is consistent with the results of Maybaum et al., who found that although LVEF significantly improved after LVAD implantation in almost all patients, longer duration of LVAD support appeared to deleteriously affect LV function [24]. Pathologic studies should be conducted to correlate galectin-3 levels with the amount of fibrosis as well as cardiac macrophage content and activation. The specific signals for increasing fibrosis after LVAD placement have not yet been fully elucidated, but the identification and reversal of this pro-fibrotic signal could assist to better define the potential of temporary LVAD placement as a “bridge to recovery” intervention in certain patients with advanced HF.

Adding to the value of galectin-3 is its potential use as a predictor of poor outcome in patients with HF. Van Kimmenade et al. found galectin-3 levels to be prognostic of adverse outcomes over 60 days [5]. Galectin-3 has also been shown to be an independent predictor of mortality in patients with NYHA class III-IV HF and in the general population, with levels >25.9 ng/ml shown to be predictive of a rapid progression of HF [3, 6, 7]. Adding to these findings, galectin-3 levels were predictive of survival in our cohort of patients undergoing LVAD placement, with plasma levels greater than 30 ng/ml at the time of implantation associated with greater mortality. Of note, although patients with galectin-3 levels >30 ng/ml were of similar age, NYHA Class, and had similar duration of HF and BNP levels than those with galectin-3 levels <30 ng//ml, patients with higher galectin-3 levels did have higher creatinine levels. It is possible, therefore, that some of the increased risk seen was due to impaired renal function. However, another study found that levels were a univariate but not independent risk factor for death in patients undergoing LVAD implantation [25]. These results indicate that this predictive value may be applicable to the subset of HF patients undergoing LVAD implantation, providing important prognostic information that may be used for patient selection and for stratifying high-risk patients.

### Galectin-3 levels following heart transplantation

We found that following HTx, the average galectin-3 level was significantly lower than in patients with severe HF. This decrease was expected with the replacement of the failing heart, but notably galectin-3 values did not return to normal levels; galectin-3 levels in transplanted patients were comparable to levels in patients with stable HF. Higher galectin-3 levels post-HTx might be related to cardiomyocyte hypertrophy and myocardial fibrosis after HTx but, of note, elevated galectin-3 levels are not related to the time period since HTx. No other study has analyzed trends in galectin-3 levels following HTx, but prior studies have shown that fibrosis increases within 2 months after HTx with little change over the subsequent 5–6 years [26]. Another explanation for dynamics in galectin-3 levels following HTx may be the use of anti-inflammatory agents to prevent rejection; as galectin-3 is an inflammatory factor released by macrophages, and its expression may be modified by this external variable.

As many as half of transplant patients will show angiographic evidence of CAV 5–15 years after HTx, accounting for 30–50 % of late graft attrition [27, 28]. Given this high prevalence and mortality, identification of CAV is important. However, diagnosis is limited by the lack of clinical symptoms of ischemia in the denervated allograft as well as limitations inherent in coronary angiography, which is a relatively insensitive study and frequently underestimates the severity and presence of CAV [29].

CAV is characterized by intimal luminal narrowing due to a fibrotic process, with a high expression of TGF-β and macrophage markers in the intima [30]. T-cells and macrophages have been shown to modulate the pathogenesis of CAV with a specific role for macrophages regulating the fibrotic response, and vascular fibrosis after HTx has been associated with more severe CAV and poor event-free survival rates [31].

Our analysis revealed a significant difference in galectin-3 levels between patients with and without CAV, and higher levels in patients with more severe degrees of CAV. Given the importance of fibrosis in CAV pathogenesis and the implication of macrophages in its development, galectin-3 may serve as a useful marker of CAV severity of. Further studies are necessary to better elucidate whether or not galectin-3 could be used as a screening tool in the diagnosis of CAV in high-risk patients, potentially linking this novel biomarker to active fibrotic processes and signaling in the cardiac allograft.

Of note, our study is limited by its retrospective observational nature with a limited number of patients from a single center. Larger, prospective studies should be done to better elucidate the value of galectin-3 as a predictive biomarker and its ability to stratify risk in HF patients, and future studies would benefit from including pathologic analysis in addition to serologic. Additionally, not all of the LVAD patients had consistent follow-up and 14 of the patients in the explant cohort did not have implant values available, which may have affected the results of the study and may represent a bias towards patients feeling well enough to come to the study visit. The small number of patients also limited our ability to correct for confounders such as age, and renal function when assessing the predictive value of galectin-3. We do not have baseline pre-HTx values for patients in the transplant cohort, limiting the direct comparison to the severe HF cohort. Additionally there are a relatively small number of patients in the CAV subgroups of post-HTx patients.

## Conclusions

Our results indicate that galectin-3 may serve as a useful biomarker for evaluating the severity and outcomes in HF and in patients undergoing LVAD implantation. Galectin-3 is an intriguing biomarker due to its relation to cardiac inflammation and fibrosis and, unlike BNP levels, galectin-3 values are not drastically changed by acute cardiac volume or pressure overload. Its role in patients following HTx needs further analysis in larger studies.

## Abbreviations

HF, heart failure; LVAD, left ventricular assist device; HTx, heart transplantation; CAV, coronary allograft vasculopathy; LVEDD, left ventricular end-diastolic diameter; LA, left atrial
